# Associations Between Neighborhood Social Cohesion and Physical Activity in the United States, National Health Interview Survey, 2017

**DOI:** 10.5888/pcd16.190085

**Published:** 2019-12-19

**Authors:** Tyler D. Quinn, Fan Wu, Dillon Mody, Brady Bushover, Dara D. Mendez, Mary Schiff, Anthony Fabio

**Affiliations:** 1 Department of Health and Physical Activity, University of Pittsburgh, Pittsburgh, Pennsylvania; 2 Department of Environmental and Occupational Health, Graduate School of Public Health, University of Pittsburgh, Pittsburgh, Pennsylvania; 3 Department of Behavioral and Community Health Sciences, Graduate School of Public Health, University of Pittsburgh, Pittsburgh, Pennsylvania; 4 Department of Epidemiology, Graduate School of Public Health, University of Pittsburgh, Pittsburgh, Pennsylvania; 5 Epidemiological Data Center, Graduate School of Public Health, University of Pittsburgh, Pittsburgh, Pennsylvania

## Abstract

**Background:**

Individual social support is positively related to physical activity participation. However, less is known about how neighborhood-level social structures relate to physical activity participation.

**Methods:**

We analyzed 2017 National Health Interview Survey data for adult participants who completed all questions on physical activity and neighborhood cohesion (N = 23,006). Each cohesion question was binary coded (cohesion or not) and used as a predictor individually and for a composite score measuring total social cohesion. We used linear regression to estimate minutes of moderate aerobic activity, and we used logistic regression to estimate the odds of meeting aerobic guidelines (≥150 min/wk), strength guidelines (≥2 d/wk of muscle strengthening exercises), or both guidelines, predicted by the 5 definitions of cohesion (composite cohesion and the 4 questions separately). Models were adjusted for sex, age, race/ethnicity, family-income-to-poverty ratio, education, nativity, language, and neighborhood tenure.

**Results:**

Respondents who reported having more social cohesion had 45.0 more minutes of aerobic activity and increased odds of meeting aerobic, strength, and combined guidelines (odds ratio [OR] = 1.22, OR = 1.13, and OR = 1.14, respectively; *P* < .01 for all). Reporting having availability of help when needed, neighbors to count on, trustworthy neighbors, and close-knit neighbors all resulted in increased odds of meeting aerobic guidelines but not increased odds for meeting strength guidelines in the latter 3 components or combined guidelines for the latter 2 components.

**Conclusions:**

Having neighborhood social cohesion or select individual components of neighborhood cohesion are positively related to meeting aerobic, strength, and combined guidelines.

**Summary**

**What is already known on this topic?**

Social support and social cohesion are strongly correlated with physical activity participation on an individual level.

**What is added by this report?**

Neighborhood-level social cohesion and its associated individual components are positively related to daily minutes of physical aerobic activity and increased odds of meeting aerobic or strength or both aerobic and strength physical activity guidelines.

**What are the implications for public health practice?**

These findings could be used to justify future experimental research on social cohesion and to identify intervention strategies focused on improving neighborhood-level social cohesion to improve physical activity behaviors of community members.

## Introduction

The 2018 Physical Activity Guidelines for Americans recommend that adults perform 150 minutes of moderate-to-vigorous aerobic physical activity per week (1,2). Meeting these recommendations has physical (eg, lower body weight, decreased incidence of type 2 diabetes and cardiovascular disease) and mental (eg, improved self-confidence and depressive symptoms) benefits (3–5). However, only 53.1% and 23.5% of people reported meeting aerobic and strength physical activity guidelines, respectively, in 2017 (6).

Social support has a strong positive relationship to duration and frequency of physical activity on an interpersonal level (eg, person-to-person interactions) (7–9). However, the socioecological model argues that physical activity is also dependent on an individual’s perception of group-level factors such as social network, neighborhood, and community (10). Evidence suggests that social support systems significantly influence physical activity self-efficacy (8). However, research has given less attention to the influence of group-level social cohesion.

The relationship between neighborhood social cohesion and physical activity is unclear. We aimed to examine the relationship between neighborhood social cohesion and its associated components and strength and aerobic physical activity in a nationally representative sample of US adults. Based on previous conclusions (11), we expected to see a positive relationship between all neighborhood social cohesion components and aerobic and strength physical activity.

## Methods

This is a secondary data analysis of 2017 National Health Interview Survey (NHIS) data from the National Center for Health Statistics (12). The NHIS is a cross-sectional household survey in which household members volunteer to participate in a face-to-face interview about their own health. This analysis was based on the 26,742 individuals in the adult subset sample (aged ≥18 y). The 2017 NHIS included 32,617 households and had a 66.5% total household response rate. The sample adult questionnaire uses a completed interview from one sample adult from each family. Of the 33,143 individuals eligible for the sample adult questionnaire, 26,742 were interviewed, resulting in a conditional response rate of 80.7%. Thus, the final unconditional sample adult response rate, considering both the family and sample adult rates, was 53.0%. More details about the NHIS are available (12).

This analysis excluded people who refused to respond or did not know the answer to any of the 3 physical activity/exercise questions (n = 1,924) or to any of the 5 neighborhood cohesion questions (n = 1,812). The final sample was N = 23,006.

### Social cohesion definitions

Neighborhood social cohesion was measured using 4 questions modified from the Project on Human Development in Chicago Neighborhoods Community Survey (13). Each participant responded to a Likert scale (1 = definitely agree, 2 = somewhat agree, 3 = somewhat disagree, and 4 = definitely disagree) in relation to the following 4 statements: 1) People in this neighborhood help each other out (help availability), 2) There are people I can count on in this neighborhood (accountability in neighbors), 3) People in this neighborhood can be trusted (trust in neighbors), and 4) This is a close-knit neighborhood (close-knit neighbors). Each score was reverse-coded so a higher score represents a higher level of agreement.

Each of the subscales was coded as having cohesion (definitely agree or somewhat agree) or not having cohesion (definitely disagree or somewhat disagree) and treated as an individual predictor. Additionally, a composite cohesion score summed the 4 subscales, resulting in a score from 4–16, with a higher score representing greater cohesion. The composite score was dichotomously coded by a median split: above median cohesion (≥13.0) or below median cohesion (<13.0), to align with previous methods (11).

### Physical activity definitions

Aerobic physical activity was based on self-reported moderate or vigorous activity. Moderate activity was defined as leisure-time periods of ≥10 continuous minutes that “cause only light sweating or a slight to moderate increase in breathing or heart rate.” Vigorous activity was defined as leisure-time periods of ≥10 continuous minutes that “cause heavy sweating or large increases in breathing or heart rate.” Moderate and vigorous aerobic activity were calculated as minutes completed per week for each. Guideline minutes/week was calculated as vigorous minutes/week multiplied by 2 and added to total moderate minutes/week (12,14). Strengthening exercises were defined as “leisure-time physical activities specifically designed to strengthen your muscles such as lifting weights or doing calisthenics” and measured in frequency per week.

In addition to the continuous variable of guideline minutes/week, 3 dichotomous physical activity variables were used for this analysis: 1) meeting aerobic guidelines, 2) meeting strength guidelines, and 3) meeting aerobic and strength guidelines. Meeting aerobic guidelines was calculated as meeting guidelines (≥150 guideline min/wk) or not (<150 guideline min/wk). Meeting strength guidelines was calculated as meeting guidelines (≥2 d/wk of strengthening exercises) or not (<2 d/wk). Meeting aerobic and strength guidelines was calculated as meeting both guidelines or not.

### Covariate definitions

All primary models included age, sex, race/ethnicity, family-income-to-poverty ratio, neighborhood tenure, education, US nativity, and English language proficiency. Age was assessed as continuous (aged 18–85 y) and then coded as 85 for anyone >85 years (11). Sex was categorized as male and female. Race/ethnicity was categorized as Hispanic, non-Hispanic white, non-Hispanic black, non-Hispanic Asian, non-Hispanic all others. Family-income-to-poverty ratio was defined as the self-reported family income divided by the poverty threshold. Neighborhood tenure was measured with the question, “About how long have you lived in your present neighborhood?” (<1 y, 1–3 y, 4–10 y, 11–20 y, >20 years, or refused/don’t know). Education was categorized as less than a high school diploma, high school diploma or equivalent, some college, college graduate, graduate degree, or refused/don’t know. Nativity was measured categorically as US born, not US born, or refused/don’t know. Self-reported English language proficiency was categorized as speaking English well, not speaking English well, or refused/don’t know.

### Analytic approach

We estimated the number of guideline minutes/week for each of the 5 cohesion definitions by using linear regression. We used logistic regression to estimate the odds ratios (ORs) of meeting the 3 types of physical activity guidelines. We used the composite cohesion score and the 4 cohesion subscales as predictors. Interaction models for meeting aerobic guidelines included interactions terms between composite cohesion and sex, race/ethnicity, family-income-to-poverty ratio, and neighborhood tenure. We used stratified analyses to examine the odds of meeting aerobic guidelines by sex, race/ethnicity, and income ratio categories. Covariates and interaction terms in the fully adjusted models were chosen based on known confounding as well as conventions and significant interactions (11). We used multiple imputation for all analyses to account for missing family income data (12). All estimates were made using the complex survey weighting and design variables described in the NHIS user guide (12). We used Stata version 14.2 (StataCorp LLC) for all analyses and set the α level at .05.

## Results

Of the total sample, slightly more than half were female (51.8%), approximately one-third were college graduates (33.0%), and most were non-Hispanic white (65.0%). The mean age of respondents was 47 years. The average family-income-to-poverty ratio was 4.25, and neighborhood tenure was evenly distributed (Table 1).

**Table 1:** Sample Characteristics, Study of the Association Between Neighborhood Social Cohesion and Physical Activity, National Health Interview Survey, United States, 2017^a^

Characteristic	Value
**Guideline min/wk (SE)**	322.5 (4.9)
**Meeting guidelines **	
Aerobic	52.9 (0.6)
Strength	27.3 (0.5)
Aerobic and strength	23.8 (0.4)
**Reported having cohesion **	
Composite cohesion	53.2 (0.6)
Help availability	83.6 (0.4)
Accountability in neighbors	83.4 (0.4)
Trust in neighbors	84.2 (0.4)
Close-knit neighbors	65.1 (0.5)
**Education **	
<High school diploma	11.7 (0.4)
High school diploma	23.5 (0.4)
Some college	18.9 (0.4)
College graduate	33.0 (0.5)
Graduate degree	12.6 (0.4)
Refused/don’t know	0.4 (0.1)
**Race/ethnicity **	
Hispanic	16.2 (0.8)
Non-Hispanic white	65.0 (0.9)
Non-Hispanic black	11.7 (0.5)
Non-Hispanic Asian	6.0 (0.3)
Non-Hispanic all other	1.1 (0.2)
**Female**	51.8 (0.4)
**Mean age, y (SE)**	47.2 (0.2)
**Family-income-to-poverty ratio (SE)**	4.2 (0.1)^b^
**Language proficiency **	
Speaks English well	94.8 (0.3)
Does not speak English well	5.2 (0.3)
Refused/don’t know	0.005 (0.004)
**Nativity **	
US-born	82.3 (0.6)
Not US-born	17.6 (0.6)
Refused/not ascertained	0.1 (0.02)
**Neighborhood tenure, y (SE)**	
<1	11.4 (0.3)
1–3	21.8 (0.4)
4–10	24.7 (0.4)
11–20	20.3 (0.4)
>20	21.7 (0.4)
Refused/don’t know	0.1 (0.02)

Abbreviation: SE, standard error.

a Data are percentages (SE) unless otherwise indicated.

b Estimated using multiple imputations.

### Neighborhood cohesion and physical activity

Estimated guideline minutes per week and the odds of meeting physical activity guidelines across composite cohesion and the 4 subscales are presented in Table 2. Compared with respondents with below median neighborhood cohesion, respondents who reported above median neighborhood cohesion had an estimated 45.0 (95% CI, 30.6–59.5) more guideline minutes/week, 1.22 (95% CI, 1.13–1.31) higher odds of meeting the aerobic guidelines, 1.13 (95% CI, 1.04–1.23) higher odds of meeting strength guidelines, and 1.14 (95% CI, 1.05–1.25) higher odds of meeting the aerobic and strength guidelines (*P* < .01 for all) (Table 2).

**Table 2:** Odds of Meeting Aerobic and Strength Guidelines, by Measures of Social Cohesion, Study of the Association Between Neighborhood Social Cohesion and Physical Activity, National Health Interview Survey, United States, 2017^a^

Variable	Guideline Min/Wk		Meeting Aerobic Guidelines^b^		Meeting Strength Guidelines^c^		Meeting Aerobic and Strength Guidelines	
	β (95% CI)	*P*	OR (95% CI)	*P*	OR (95% CI)	*P*	OR (95% CI)	*P*
Composite cohesion	45.0 (30.6–59.5)	<.001	1.22 (1.13–1.31)	<.001	1.13 (1.04–1.23)	.005	1.14 (1.05–1.25)	.003
Help availability	40.9 (21.4–60.4)	<.001	1.35 (1.22–1.50)	<.001	1.26 (1.09–1.45)	.001	1.26 (1.09–1.45)	.002
Accountability in neighbors	48.3 (28.8–67.9)	<.001	1.35 (1.22–1.50)	<.001	1.14 (0.99–1.32)	.07	1.17 (1.01–1.35)	.03
Trust in neighbors	35.0 (14.7–55.3)	.001	1.32 (1.19–1.46)	<.001	1.13 (0.99–1.30)	.08	1.13 (0.98–1.31)	.08
Close-knit neighbors	40.1 (25.1–55.0)	<.001	1.17 (1.08–1.25)	<.001	1.08 (0.99–1.18)	.09	1.08 (0.98–1.17)	.11

Abbreviations: CI, confidence interval; OR, odds ratio.

a All models were adjusted for neighborhood tenure, sex, race/ethnicity, income, age, education, nativity, and language proficiency.

b Aerobic physical activity was based on self-reported moderate (defined as leisure-time periods of ≥10 continuous minutes that cause only light sweating or a slight to moderate increase in breathing or heart rate) or vigorous activity (defined as leisure-time periods of ≥10 continuous minutes that cause heavy sweating or large increases in breathing or heart rate). Minutes of vigorous activity were multiplied by 2 and added to the number of minutes of moderate activity, and meeting aerobic guidelines was defined as ≥150 min/wk.

c Strengthening exercises were defined as leisure-time physical activities specifically designed to strengthen muscles (eg, lifting weights, doing calisthenics) and measured in frequency per week. Meeting strength guidelines was defined as ≥2 days/week of strengthening exercises.

Help availability was significantly associated with higher odds of achieving all guidelines (Table 2). Accountability in neighbors was significantly associated with higher odds of meeting aerobic guidelines (OR, 1.35; 95% CI, 1.22–1.50) and of meeting aerobic and strength guidelines in combination (OR, 1.17; 95% CI, 1.01–1.35) but not with meeting strength guidelines alone. Trust in neighbors (OR = 1.32; 95% CI, 1.19–1.46) and having close-knit neighbors (OR, 1.17; 95% CI, 1.08–1.25) were significantly associated with higher odds of meeting aerobic guidelines only (Table 2). 

### Interactions

A significant interaction between social cohesion and sex was found in meeting aerobic (*P* = .025) and strength (*P* = .005) guidelines. In stratified analyses, both sexes had significantly increased odds of meeting aerobic guidelines with above median social cohesion (*P* < .001 for both). However only female respondents had significantly increased odds of meeting strength guidelines with above median cohesion scores (Table 3).

**Table 3:** Odds of Meeting Aerobic and Strength Guidelines, by Demographic Variables, Study of the Association Between Neighborhood Social Cohesion and Physical Activity, National Health Interview Survey, United States, 2017^a^

Variable	Meeting Aerobic Guidelines^b^		Meeting Strength Guidelines^c^	
	OR (95% CI)	*P* Value	OR (95% CI)	*P* Value
**Sex**	**Overall *P* = .025**		**Overall *P* = .005**	
Male	1.04 (1.02–1.05)	<.001	1.00 (0.98–1.03)	.70
Female	1.05 (1.03–1.06)	<.001	1.04 (1.02–1.06)	<.001
**Race/ethnicity**	**Overall *P* = .57**		**Overall *P* = .67**	
Hispanic	1.04 (1.01–1.07)	.008	1.02 (0.97–1.06)	.47
Non-Hispanic white	1.05 (1.04–1.07)	<.001	1.03 (1.01–1.04)	<.001
Non-Hispanic black	1.00 (0.97–1.04)	.80	1.01 (0.97–1.07)	.56
Non-Hispanic Asian	1.02 (0.97–1.07)	.47	0.99 (0.93–1.05)	.71
Non-Hispanic all other	1.08 (0.97–1.20)	.17	1.01 (0.92–1.11)	.80
**Family-income-to-poverty ratio**	**Overall *P* = .02**		**Overall *P* = .21**	
Ratio ≤1.0	1.08 (1.04–1.11)	<.001	1.07 (1.02–1.12)	.006
Ratio >1.0	1.05 (1.03–1.06)	<.001	1.02 (1.01–1.04)	.004
**Neighborhood tenure**	**Overall *P* = .41**		**Overall *P* = .50**	

Abbreviations: CI, confidence interval; OR, odds ratio.

a Overall group *P* values represent significance of the overall interaction term. All data are for respondents who reported above median social cohesion.

b Aerobic physical activity was based on self-reported moderate (defined as leisure-time periods of ≥10 continuous minutes that cause only light sweating or a slight to moderate increase in breathing or heart rate) or vigorous activity (defined as leisure-time periods of ≥10 continuous minutes that cause heavy sweating or large increases in breathing or heart rate). Minutes of vigorous activity were multiplied by 2 and added to the number of minutes of moderate activity, and meeting aerobic guidelines was defined as ≥150 min/wk.

c Strengthening exercises were defined as leisure-time physical activities specifically designed to strengthen muscles (eg, lifting weights, doing calisthenics) and measured in frequency per week. Meeting strength guidelines was defined as ≥2 days/week of strengthening exercises.

No significant interaction was found between composite cohesion and race/ethnicity in either meeting aerobic (*P* = 0.57) or strength (*P* = .67) guidelines. However, in stratified analyses, white respondents who reported above median neighborhood cohesion had higher odds of meeting aerobic guidelines (OR = 1.05; 95% CI, 1.04–1.07) and strength guidelines (OR = 1.03; 95% CI, 1.01–1.04). Hispanic individuals who reported above median social cohesion had higher odds of meeting aerobic guidelines (OR = 1.04, 95% CI, 1.01–1.07) (Table 3).

The interaction between composite cohesion and family-income-to-poverty ratio was significant for meeting aerobic (*P* = .02) but not strength (*P* = .21) guidelines. Stratified analyses showed no difference in meeting either guideline in those above or below a ratio of 1.0 (Table 3). The interaction between neighborhood tenure and composite cohesion was not significant in meeting either aerobic (*P* = .41) or strength (*P* = .50) guidelines.

## Discussion

We analyzed data from a nationally representative sample to determine whether perceived neighborhood cohesion has a positive relationship with meeting aerobic and strength physical activity recommendations. The magnitude of the effects was similar for most components of neighborhood cohesion; however, the effects on strength activities were weaker overall. Interaction effects were observed across categories of sex in aerobic and strength guidelines and by family-income-to-poverty ratio in aerobic only. No interactions were observed by race/ethnicity or neighborhood tenure.

These results are congruent with those from similar work that used 2013–2014 NHIS data and found an adjusted odds ratio of 1.22 between dichotomized total neighborhood cohesion and meeting aerobic physical activity guidelines (11). Our findings, using the 2017 NHIS data, found similar and significantly higher odds for meeting aerobic (OR = 1.22), strength (OR = 1.13), and combined recommendations (OR = 1.14). Both studies are supported by findings of previous literature, indicating that social support is a strong determinant of physical activity participation (15). Social cohesion and safety are associated with increased odds of walking (OR = 1.78; 95% CI, 1.56–2.03) and moderate physical activity (OR = 1.93; 95% CI, 1.65–2.27) in a population-based study in the United Kingdom (16). Our findings extend those results to strength training specifically.

Previous work demonstrated that increased neighborhood social cohesion and trust can increase the sense of safety and shared goals toward physical activity (17,18). Yi et al examined the relationship between total neighborhood social cohesion and meeting aerobic physical activity recommendations using the 2013–2014 NHIS (11). They found significantly increased odds of meeting aerobic physical activity recommendations with higher levels of neighborhood social cohesion, with stronger associations in non-Hispanic whites (11). Similarly, a study of Canadian adults found that community social cohesion is positively associated with physical activity (19). However, Lindstrom et al found that individuals reporting more social participation (a potential product of high social cohesion) have less reported physical activity (20). Although some research exists, further investigations should identify specific components of social cohesion that influence self-reported strength and aerobic physical activity.

This study adds to the literature by investigating effects of subscales of social cohesion. The adjusted ORs for the 4 cohesion components were all moderately significantly positive in relation to aerobic guidelines. Perceiving one’s neighborhood as close-knit resulted in slightly lower odds of meeting any definition of the guidelines than did the other 3 components. This finding could be because the motivation for physical activity participation in a neighborhood is partially driven by perceived neighborhood safety (21), which is potentially supported by trusting and accountable neighbors. Having close-knit neighbors could be a component of cohesion more related to physical (in-person) support for physical activity that is likely to be more sporadic in nature and therefore does not support as strongly activity in line with the guidelines. Although confirmation studies are still required, the distinction made in this study between social cohesion and its related components is important for social programs to influence activity. Focusing on individual cohesion components may be appropriate.

We included strength guidelines, which provided an addition to the literature. We found less consistent results in meeting strength than aerobic guidelines, where 3 cohesion components did not show significantly different odds in meeting strength guidelines and 2 were nonsignificant in the combined guidelines. Although subjective norms influence strength training participation (22), this is the first study to examine this relationship with neighborhood-level social cohesion and meeting strength training guidelines. Without literature to compare with, we hypothesize that strength exercise may be less influenced than aerobic exercise by social cohesion, which could be due to the ease of socialization during aerobic exercise compared with strength exercise. Furthermore, strength training participation is higher among men than women (23), although in this analysis women showed a stronger positive relationship between social cohesion and physical activity participation than men. This sex difference may explain some of the lower overall effect of social cohesion on meeting strength guidelines. However, why the effects of social cohesion differ by aerobic and strength activities remains unclear, and further research is warranted.

This analysis also explored interactions with demographic factors. We found a significant sex-by-cohesion interaction for both aerobic and strength guidelines. Although the interaction for sex was statistically significant (possibly because of the large sample size), the magnitude of difference between the odds among men and women was low (<.05). However, women had slightly higher odds of meeting aerobic recommendations if they perceive having above median neighborhood cohesion, whereas men did not. Perceptions of neighborhood safety influence physical activity, especially in women (21). It is possible that increased neighborhood social cohesion increases perception of safety among women. Furthermore, the interaction by sex in meeting strength guidelines was stronger in women than men. This is the first study, to our knowledge, where this has been explored.

We found a significant interaction between family-income-to-poverty ratio and cohesion with meeting aerobic guidelines. However, similar to race, a stratified analysis of families above and below the poverty threshold found no meaningful difference in magnitudes of effects. However, it is plausible that the slightly higher odds seen in those below the poverty threshold may be related to the feeling of safety in those neighborhoods where people of lower income rely on their neighbors to feel safe enough to be active.

There was no significant interaction between race/ethnicity and cohesion in either strength or aerobic guidelines. When stratifying the analysis by racial/ethnic groups, significant positive odds for meeting aerobic and strength guidelines were found in non-Hispanic whites and in Hispanics for aerobic only but no other racial/ethnic group, which is consistent with previous results reporting higher odds of meeting aerobic guidelines in non-Hispanic whites (11). Differences by race/ethnicity and income level may be due to differences in resource availability (ie, institutionalized racism) (24,25), social capital (8), and adverse neighborhood environments (26). Furthermore, the positive effect of social cohesion on physical activity behaviors depends on interpersonal relationships and observed physical activity patterns of an individual’s peers and community members (8,27). Many neighborhoods are racially segregated in the United States, and leisure-time physical activity is lower in African American and Hispanic populations compared with whites (28). Therefore, neighborhood social cohesion among certain black, Hispanic, and low-income populations, for example, may not support physical activity in the same way as among white or higher-income populations.

Our study has strengths. The large, nationally representative sample make these results generalizable to US residents. In addition, we used the subscale scores as well as an aggregate cohesion score, which demonstrated that social cohesion was a combination of perceived features of communities. Lastly, self-reported physical activity was estimated with both strength training guidelines and aerobic guidelines, whereas previous analyses used aerobic guidelines only.

Our study also has limitations. We used cross-sectional data, so temporality or causality cannot be established. Additionally, data for perceived neighborhood cohesion and physical activity were all self-reported and are subject to reporting and recall biases. As such, over-reporting of cohesion and physical activity would likely result in bias away from the null because the over-reporting in both questions may come from the same individuals. Moreover, perceived data are subject to differing individual definitions for the social cohesion components. However, we were not able to identify or adjust for that. Lastly, the 2017 NHIS used the definition of aerobic physical activity as ≥10-minute bouts whereas the 2018 guidelines recommended bouts of any duration. This difference in definition limits this analysis in future comparisons.

More studies should examine neighborhood cohesion and the duration and type of physical activity. Experimental or longitudinal studies should also explore the potential effects of neighborhood-level social programming on physical activity behaviors. Also, multilevel or clustered data analysis could be used to investigate the influence of household clusters, town/city, or county levels. Lastly, objective measurement of physical activity (eg, accelerometry) should further confirm these results.

This study confirmed and expanded on previous literature to show that neighborhood social cohesion is positively associated with physical activity participation. A modest yet significant increase in odds for meeting aerobic, strength, and combined guidelines was found in individuals reporting above median perceived neighborhood social cohesion. This relationship should be further explored using experimental or longitudinal designs to further understand a potential causal relationship, and our results can be used to motivate such future work.

## References

[1] Arem H , Moore SC , Patel A , Hartge P , Berrington de Gonzalez A , Visvanathan K , Leisure time physical activity and mortality: a detailed pooled analysis of the dose-response relationship. JAMA Intern Med 2015;175(6):959–67. 10.1001/jamainternmed.2015.0533 25844730 PMC4451435

[2] Centers for Disease Control and Prevention. 2018 Physical activity guidelines for Americans. Washington (DC): US Department of Health and Human Services; 2018.

[3] Hills AP , Dengel DR , Lubans DR . Supporting public health priorities: recommendations for physical education and physical activity promotion in schools. Prog Cardiovasc Dis 2015;57(4):368–74. 10.1016/j.pcad.2014.09.010 25269062

[4] Friedenreich CM , Orenstein MR . Physical activity and cancer prevention: etiologic evidence and biological mechanisms. J Nutr 2002;132(11 Suppl):3456S–64S. 10.1093/jn/132.11.3456S 12421870

[5] Artinian NT , Fletcher GF , Mozaffarian D , Kris-Etherton P , Van Horn L , Lichtenstein AH , ; American Heart Association Prevention Committee of the Council on Cardiovascular Nursing. Interventions to promote physical activity and dietary lifestyle changes for cardiovascular risk factor reduction in adults: a scientific statement from the American Heart Association. Circulation 2010;122(4):406–41. 10.1161/CIR.0b013e3181e8edf1 20625115 PMC6893884

[6] Centers for Disease Control and Prevention. Early release of selected estimates based on data from the 2017 National Health Interview Survey, data tables for figures 7.1, 7.5. Atlanta (GA): Centers for Disease Control and Prevention; 2017.

[7] Kahn EB , Ramsey LT , Brownson RC , Heath GW , Howze EH , Powell KE , The effectiveness of interventions to increase physical activity. A systematic review. Am J Prev Med 2002;22(4 Suppl):73–107. 10.1016/S0749-3797(02)00434-8 11985936

[8] McNeill LH , Kreuter MW , Subramanian SV . Social environment and physical activity: a review of concepts and evidence. Soc Sci Med 2006;63(4):1011–22. 10.1016/j.socscimed.2006.03.012 16650513

[9] Sternfeld B , Ainsworth BE , Quesenberry CP Jr . Physical activity patterns in a diverse population of women. Prev Med 1999;28(3):313–23. 10.1006/pmed.1998.0470 10072751

[10] Giles-Corti B , Donovan RJ . The relative influence of individual, social and physical environment determinants of physical activity. Soc Sci Med 2002;54(12):1793–812. 10.1016/S0277-9536(01)00150-2 12113436

[11] Yi SS , Trinh-Shevrin C , Yen IH , Kwon SC . Racial/ethnic differences in associations between neighborhood social cohesion and meeting physical activity guidelines, United States, 2013–2014. Prev Chronic Dis 2016;13:E160261. 10.5888/pcd13.160261 27930284 PMC5145691

[12] Survey description, National Health Interview Survey, 2017. Hyattsville (MD): National Center for Health Statistics; 2018.

[13] Sampson RJ , Raudenbush SW , Earls F . Neighborhoods and violent crime: a multilevel study of collective efficacy. Science 1997;277(5328):918–24. 10.1126/science.277.5328.918 9252316

[14] Centers for Disease Control and Prevention. 2008 Physical activity guidelines for Americans. Atlanta (GA): Centers for Disease Control and Prevention; 2008.

[15] Trost SG , Owen N , Bauman AE , Sallis JF , Brown W . Correlates of adults’ participation in physical activity: review and update. Med Sci Sports Exerc 2002;34(12):1996–2001. 10.1097/00005768-200212000-00020 12471307

[16] Sawyer ADM , Jones R , Ucci M , Smith L , Kearns A , Fisher A . Cross-sectional interactions between quality of the physical and social environment and self-reported physical activity in adults living in income-deprived communities. PLoS One 2017;12(12):e0188962. 10.1371/journal.pone.0188962 29240791 PMC5730220

[17] Ross CE , Jang SJ . Neighborhood disorder, fear, and mistrust: the buffering role of social ties with neighbors. Am J Community Psychol 2000;28(4):401–20. 10.1023/A:1005137713332 10965384

[18] Bazaco MC , Pereira MA , Wisniewski SR , Zgibor JC , Songer TJ , Burke JD , Is there a relationship between perceived neighborhood contentedness and physical activity in young men and women? J Urban Health 2016;93(6):940–52. 10.1007/s11524-016-0088-z 27798762 PMC5126024

[19] Yip C , Sarma S , Wilk P . The association between social cohesion and physical activity in Canada: a multilevel analysis. SSM Popul Health 2016;2:718–23. 10.1016/j.ssmph.2016.09.010 29349183 PMC5757883

[20] Lindström M , Hanson BS , Ostergren PO . Socioeconomic differences in leisure-time physical activity: the role of social participation and social capital in shaping health related behaviour. Soc Sci Med 2001;52(3):441–51. 10.1016/S0277-9536(00)00153-2 11330778

[21] Carlson JA , Bracy NL , Sallis JF , Millstein RA , Saelens BE , Kerr J , Sociodemographic moderators of relations of neighborhood safety to physical activity. Med Sci Sports Exerc 2014;46(8):1554–63. 10.1249/MSS.0000000000000274 25029166 PMC4101912

[22] Dean RN , Farrell JM , Kelley ML , Taylor MJ , Rhodes RE . Testing the efficacy of the theory of planned behavior to explain strength training in older adults. J Aging Phys Act 2007;15(1):1–12. 10.1123/japa.15.1.1 17387225

[23] Centers for Disease Control and Prevention. Prevention: trends in strength training — United States, 1998–2004. MMWR Morb Mortal Wkly Rep 2006;55(28):769–72. 16855525

[24] Boslaugh SE , Luke DA , Brownson RC , Naleid KS , Kreuter MW . Perceptions of neighborhood environment for physical activity: is it “who you are” or “where you live”? J Urban Health 2004;81(4):671–81. 10.1093/jurban/jth150 15466848 PMC3455921

[25] King AC , Jeffery RW , Fridinger F , Dusenbury L , Provence S , Hedlund SA , Environmental and policy approaches to cardiovascular disease prevention through physical activity: issues and opportunities. Health Educ Q 1995;22(4):499–511. 10.1177/109019819502200407 8550373

[26] Seefeldt V , Malina RM , Clark MA . Factors affecting levels of physical activity in adults. Sports Med 2002;32(3):143–68. 10.2165/00007256-200232030-00001 11839079

[27] Ståhl T , Rütten A , Nutbeam D , Bauman A , Kannas L , Abel T , The importance of the social environment for physically active lifestyle—results from an international study. Soc Sci Med 2001;52(1):1–10. 10.1016/S0277-9536(00)00116-7 11144909

[28] He XZ , Baker DW . Differences in leisure-time, household, and work-related physical activity by race, ethnicity, and education. J Gen Intern Med 2005;20(3):259–66. 10.1111/j.1525-1497.2005.40198.x 15836530 PMC1490074

